# The socket-shield technique: a critical literature review

**DOI:** 10.1186/s40729-020-00246-2

**Published:** 2020-09-07

**Authors:** Christian Blaschke, Donald R. Schwass

**Affiliations:** 1 Department of Oral Diagnostic and Surgical Sciences, Faculty of Dentistry, University of Otago, 310 Great King Street, Dunedin, New Zealand; 2 Faculty of Dentistry, University of Otago, 310 Great King Street, Dunedin, New Zealand

**Keywords:** Dental implants, Socket-shield, Root-membrane, Partial extraction, Bone preservation, Root submersion

## Abstract

**Introduction:**

Dental implants have become a standard treatment in the replacement of missing teeth. After tooth extraction and implant placement, resorption of buccal bundle bone can pose a significant complication with often very negative cosmetic impacts. Studies have shown that if the dental root remains in the alveolar process, bundle bone resorption is very minimal. However, to date, the deliberate retention of roots to preserve bone has not been routinely used in dental implantology.

**Material and methods:**

This study aims to collect and evaluate the present knowledge with regard to the socket-shield technique as described by Hurzeler et al. (J Clin Periodontol 37(9):855-62, 2010). A PubMed database search (www.ncbi.nlm.nih.gov/pubmed) was conducted to identify relevant publication.

**Results:**

The initial database search returned 229 results. After screening the abstracts, 13 articles were downloaded and further scrutinised. Twelve studies were found to meet the inclusion and exclusion criteria.

**Conclusion:**

Whilst the socket-shield technique potentially offers promising outcomes, reducing the need for invasive bone grafts around implants in the aesthetic zone, clinical data to support this is very limited. The limited data available is compromised by a lack of well-designed prospective randomised controlled studies. The existing case reports are of very limited scientific value. Retrospective studies exist in limited numbers but are of inconsistent design. At this stage, it is unclear whether the socket-shield technique will provide a stable long-time outcome.

## Introduction

**Fig. 1:**
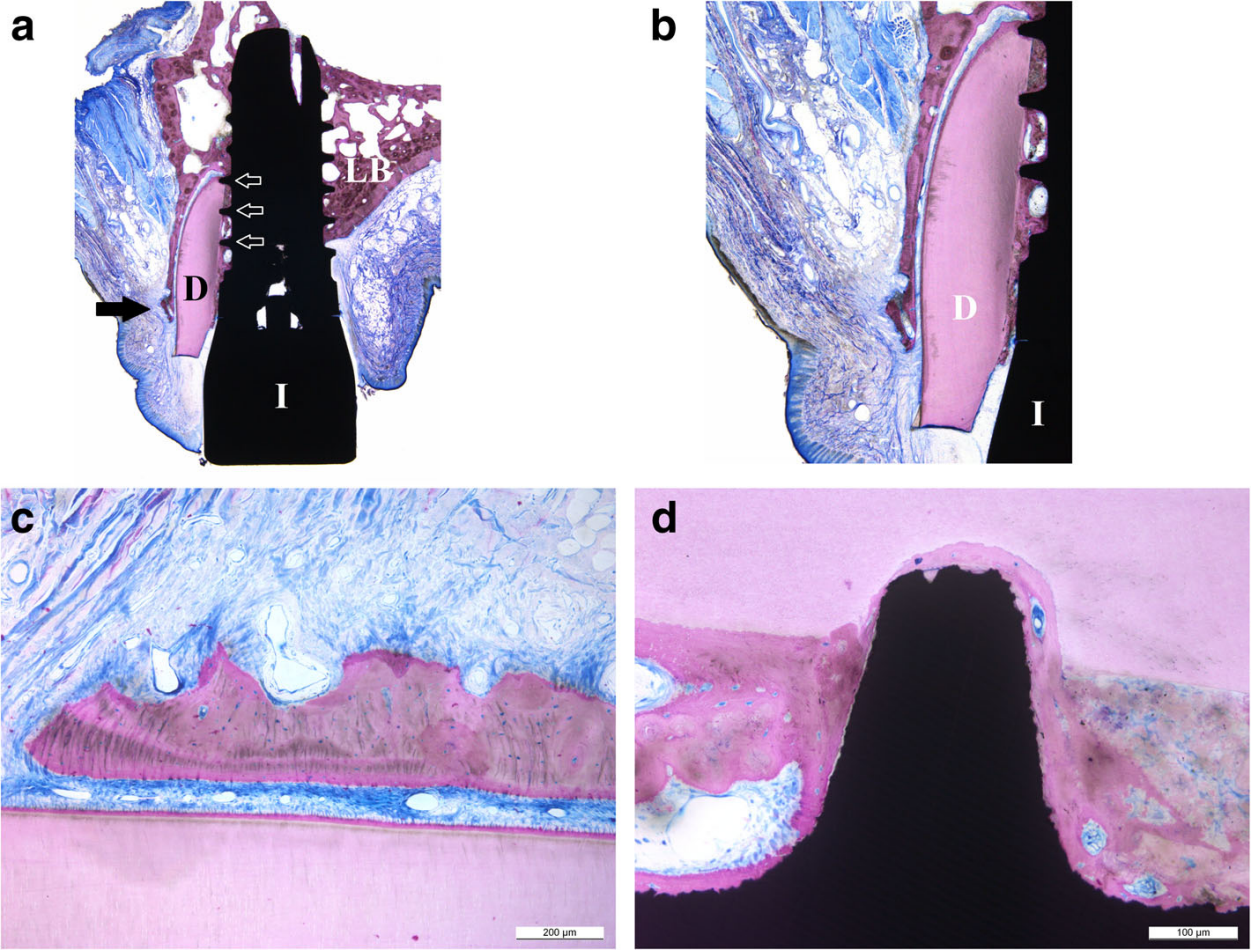
**a**–**d** Histologies of Beagle dog socket shields

Dental implants have become a standard treatment in the replacement of missing teeth. Whilst initially dental implants were mainly used to secure complex multi-unit prostheses, in recent decades, it has become common to replace single teeth, in particular in the aesthetic zone. Paired with the ever increasing demand to achieve cosmetically pleasing outcomes, this has led to the demand to preserve buccal hard and soft tissues. After tooth extraction and implant placement, resorption of buccal bundle bone can pose a significant complication with often very negative cosmetic impacts. Hence, grafting procedures are commonly carried out with the intention of minimising loss of bundle bone. However, if it proved possible to preserve bundle bone, these graft procedures might not be necessary. Studies have shown that if the dental root remains in the alveolar process, bundle bone resorption is very minimal. Knowing this, the technique of retaining roots has long been utilised for cases involving removable prostheses, and to a lesser degree, fixed prostheses. However, to date, the deliberate retention of roots to preserve bone has not been routinely used in dental implantology. Back as early as 2010, Hurzeler et al. published a proof of concept proposing partial retention of tooth roots in an effort to preserve the important buccal bone. Preservation of bone and ossification between residual roots and surrounding bone have been demonstrated in beagle dogs [1] (Fig. 1a–d histology of socket-shield in beagle dogs).

Hurzeler et al. postulated that leaving a 1.5-mm-thick root fragment on the buccal aspect of the proposed implant site [1] would leave sufficient space for optimal placement of the dental implant as well as maintain the buccal plate.

Figures 2, 3, 4, 5, 6, 7, 8, 9, 10, 11, 12, and 13 illustrate the socket-shield technique as per Hurzeler et al.

**Fig. 2:**
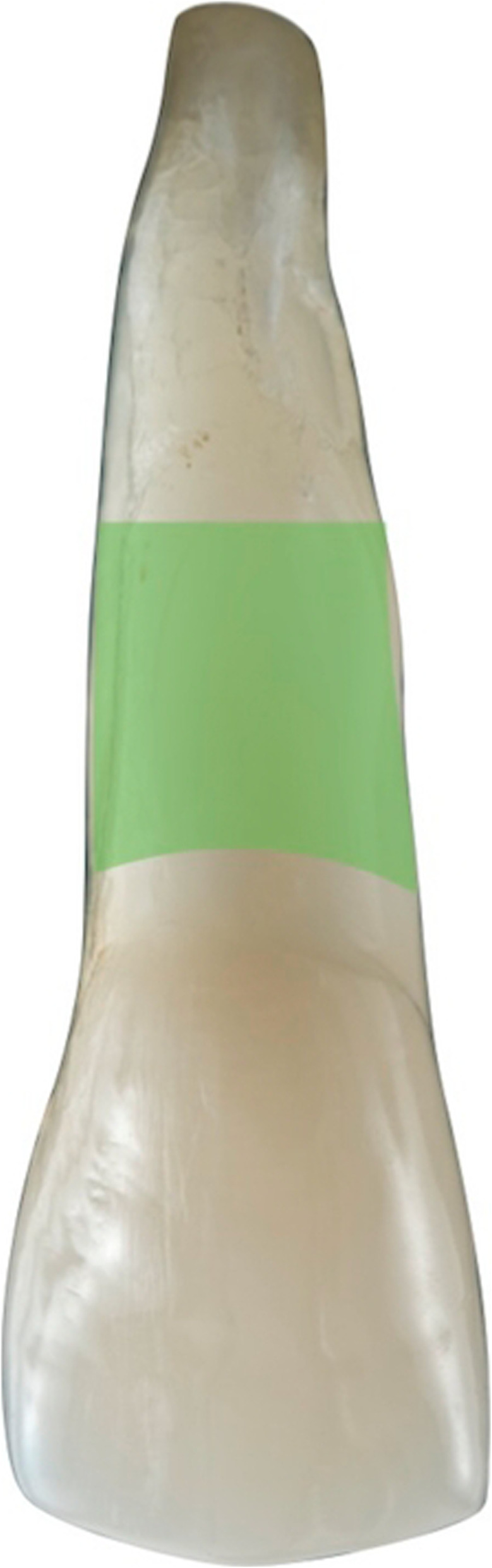
Socket-shield schematic, remaining root section (facial view)

**Fig. 3:**
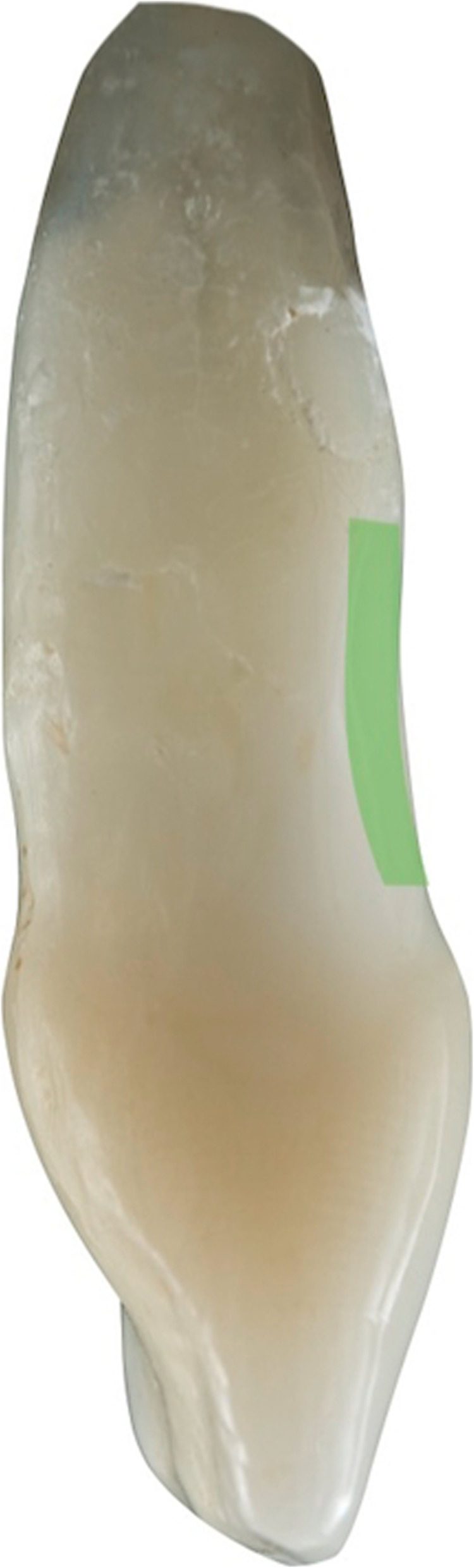
Socket-shield schematic, remaining root section (transverse view)

**Fig. 4:**
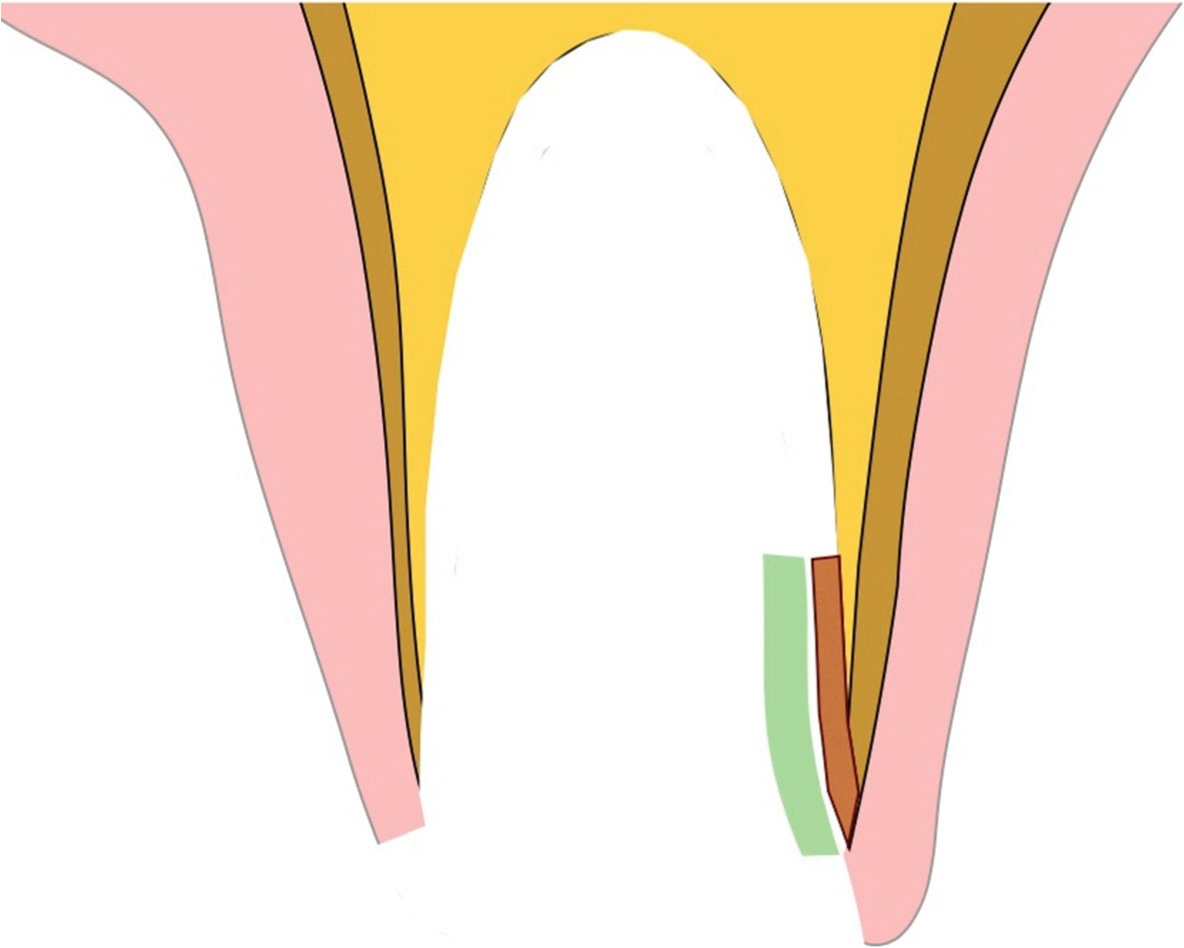
Socket-shield schematic (transverse view)

**Fig. 5:**
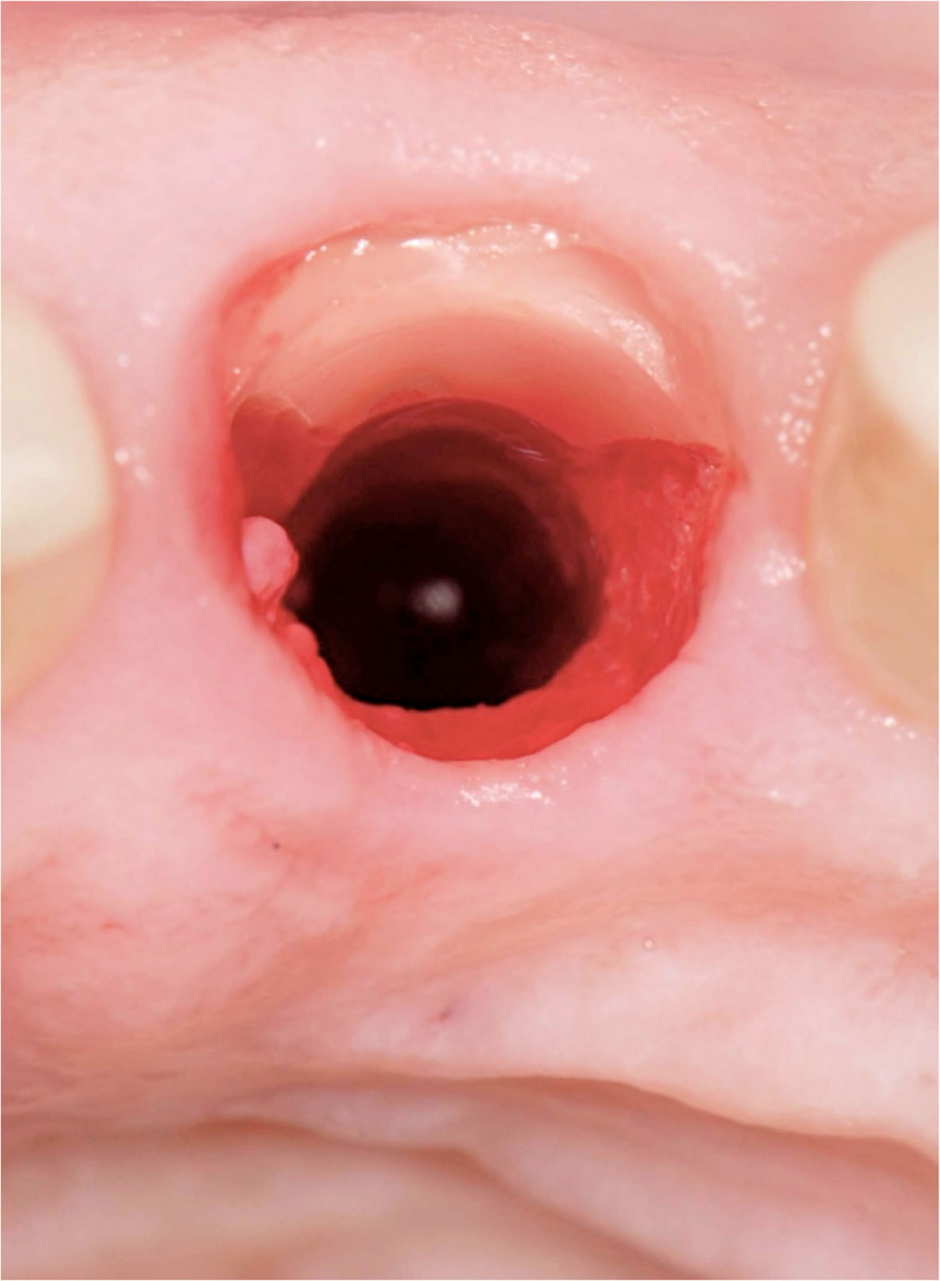
Socket-shield in vivo (occlusal view)

**Fig. 6:**
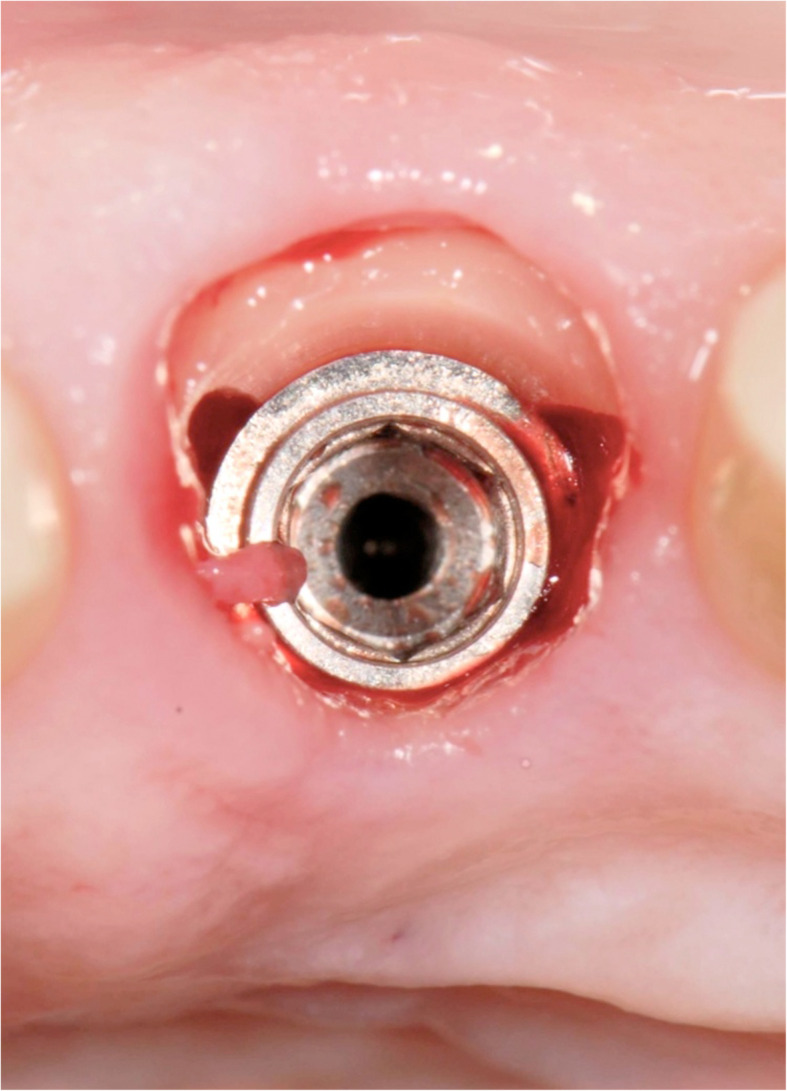
Implant placed palatally to socket shield

**Fig. 7:**
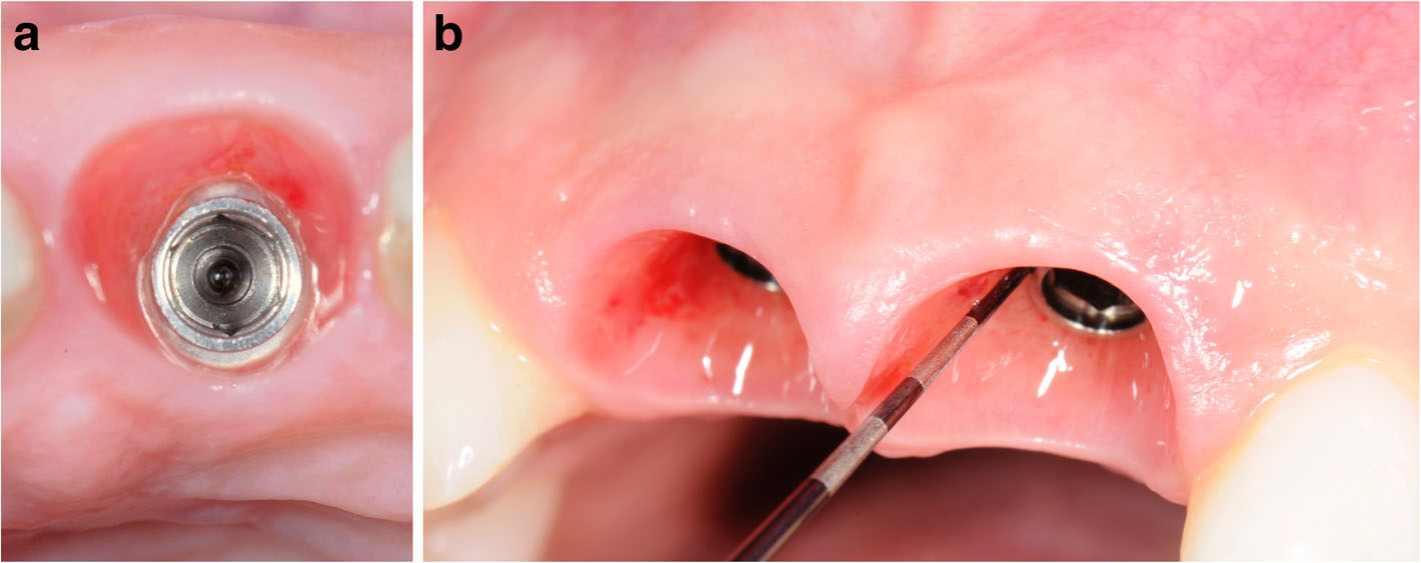
**a** Healed implant site (occlusal view). **b** Healed implant site, emergence profile

**Fig. 8:**
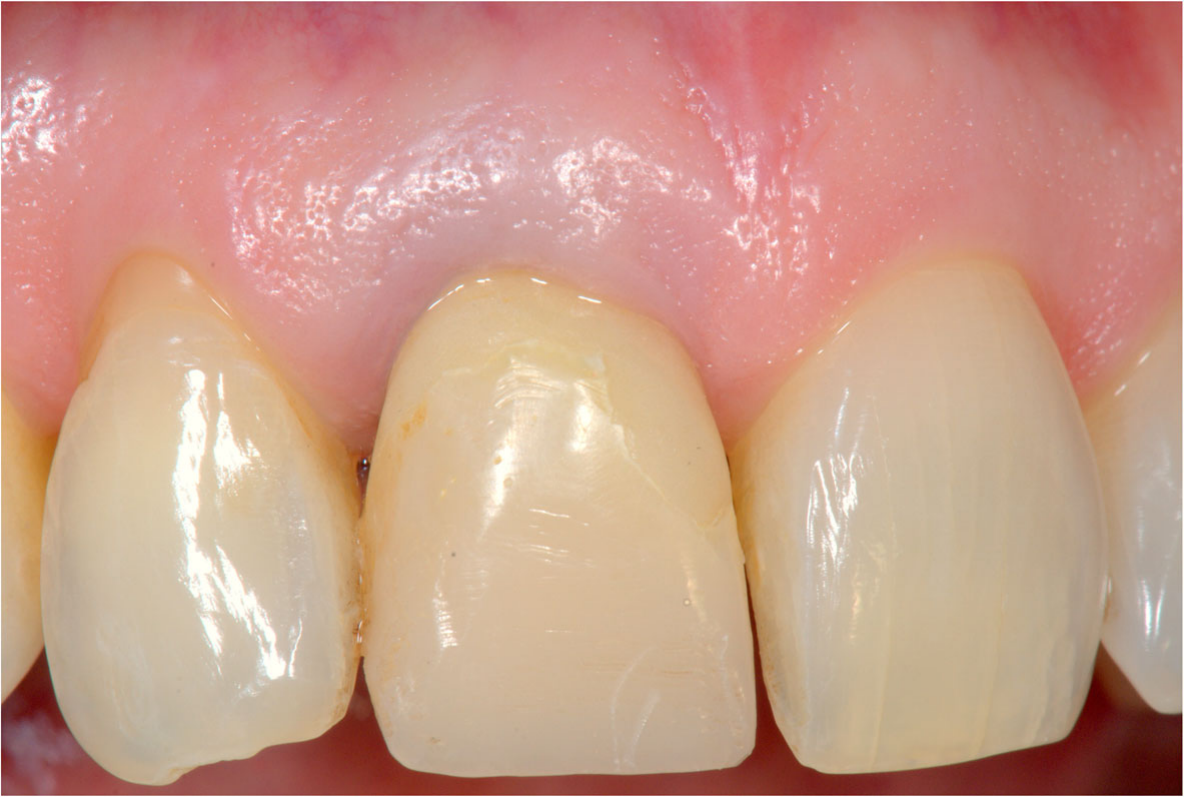
Preoperative tooth (facial view)

**Fig. 9:**
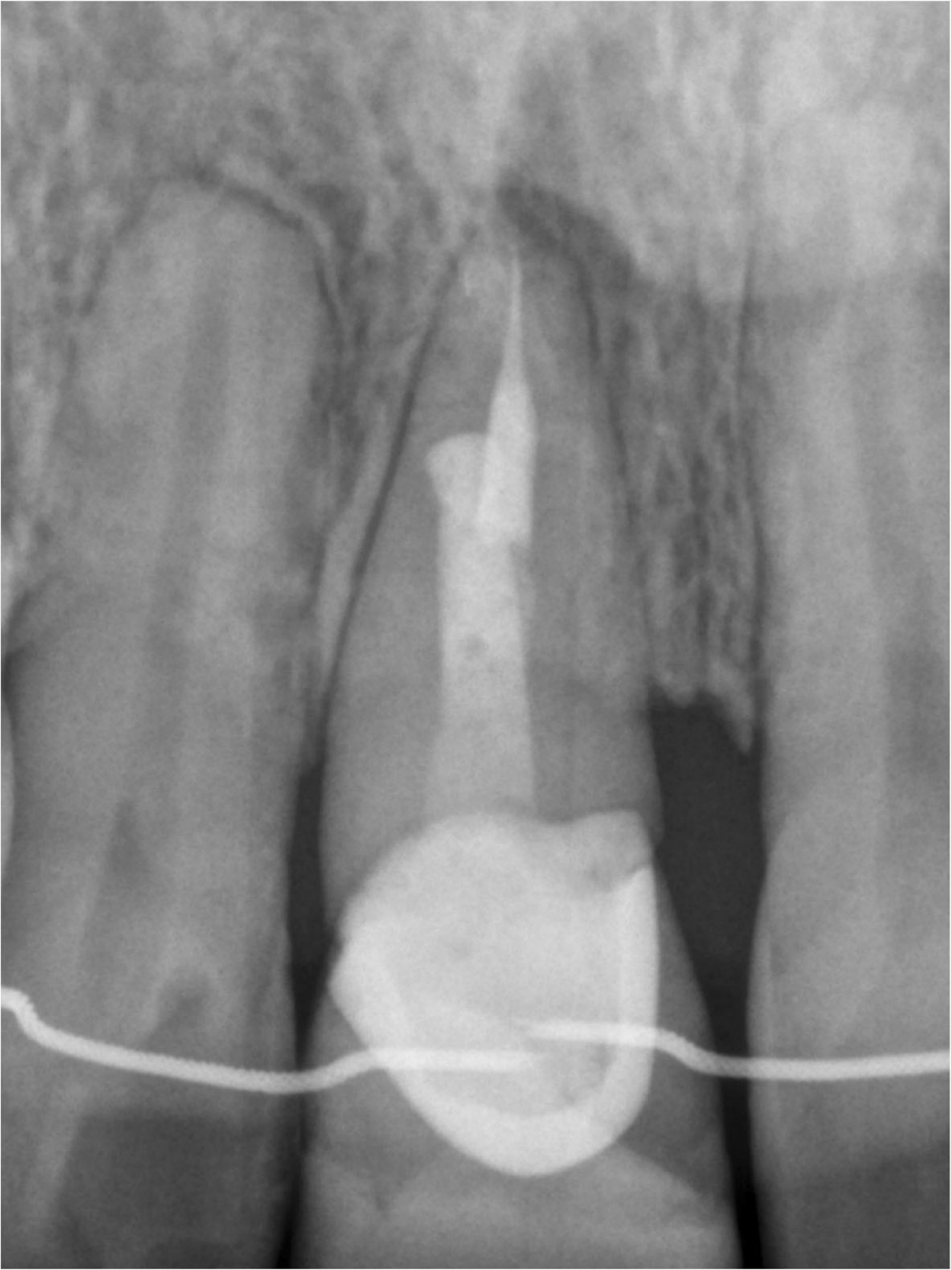
Preoperative x-ray

**Fig. 10:**
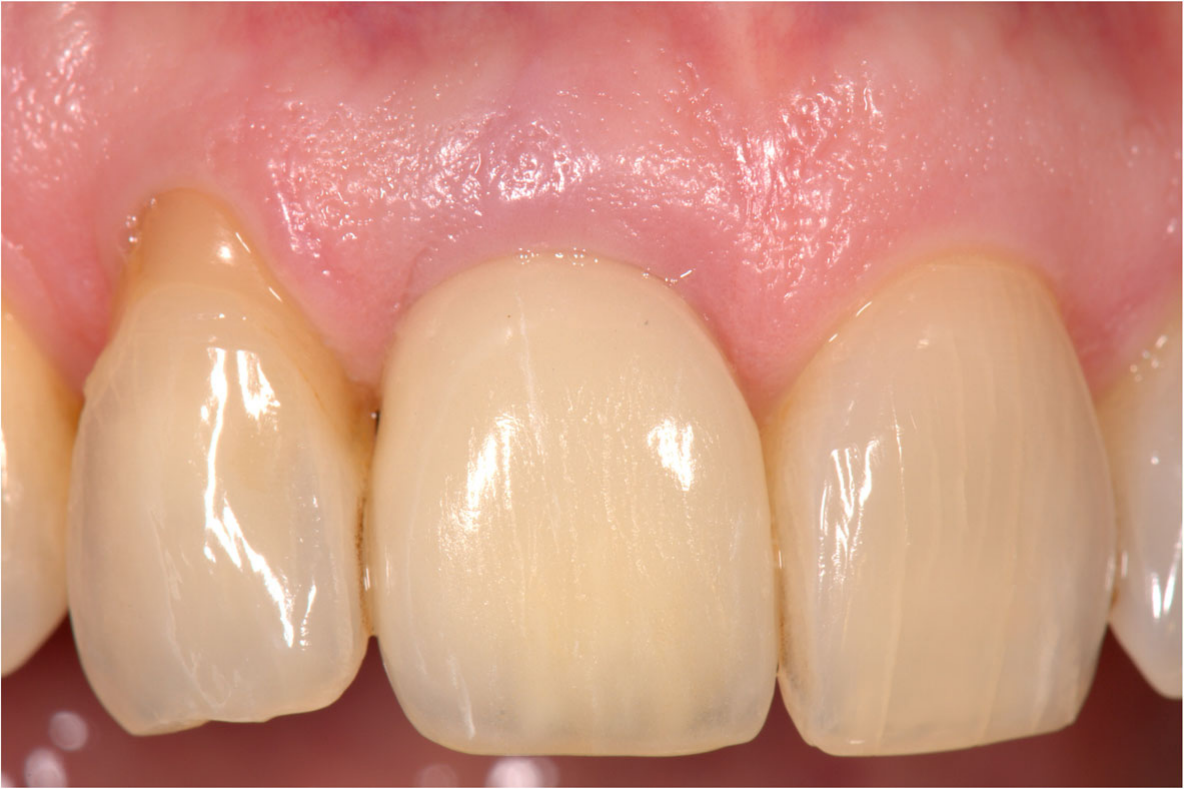
Implant restoration in situ (facial view)

**Fig. 11:**
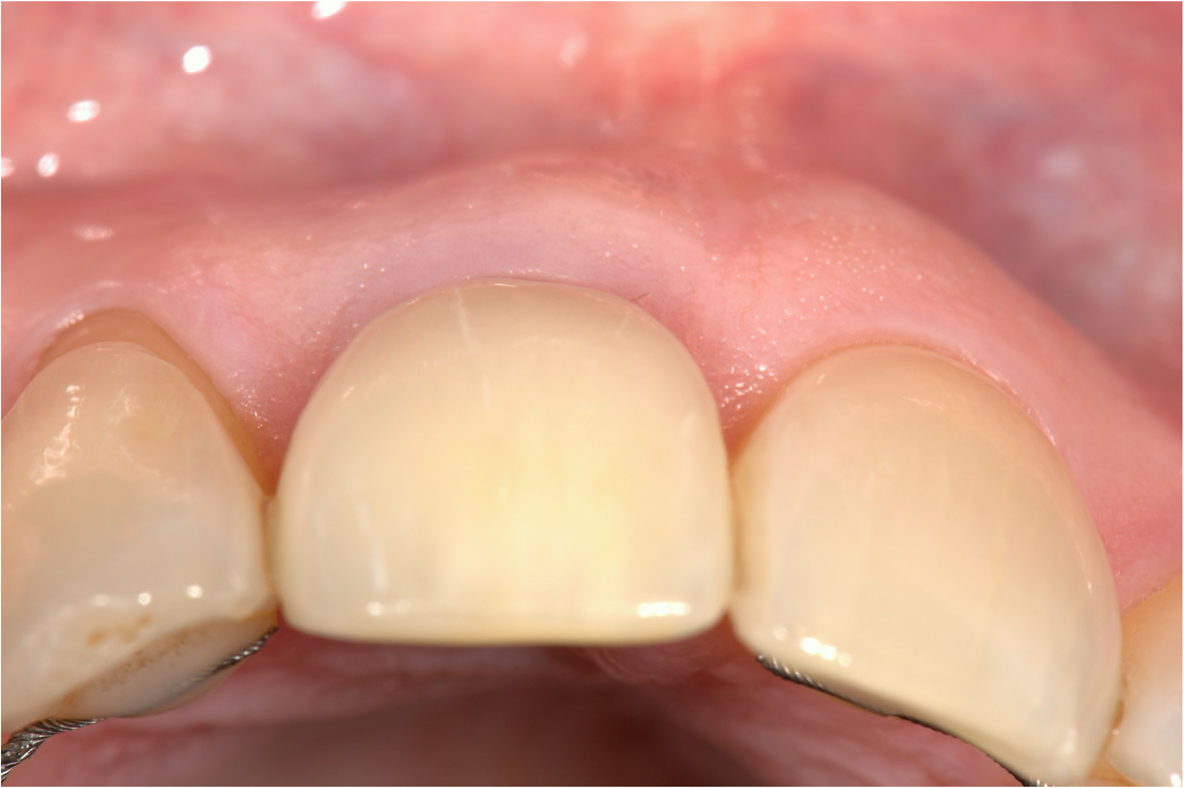
Implant restoration in situ (occlusal view)

**Fig. 12:**
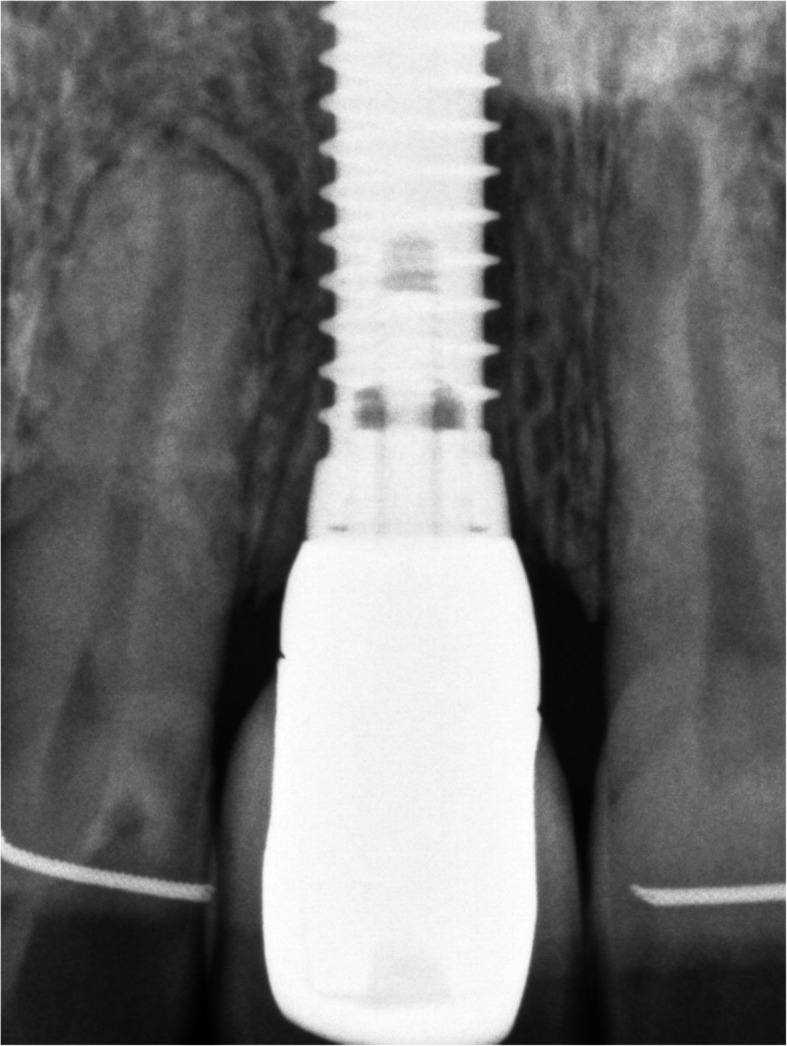
Postoperative x-ray at time of fitting of implant placement

**Fig. 13:**
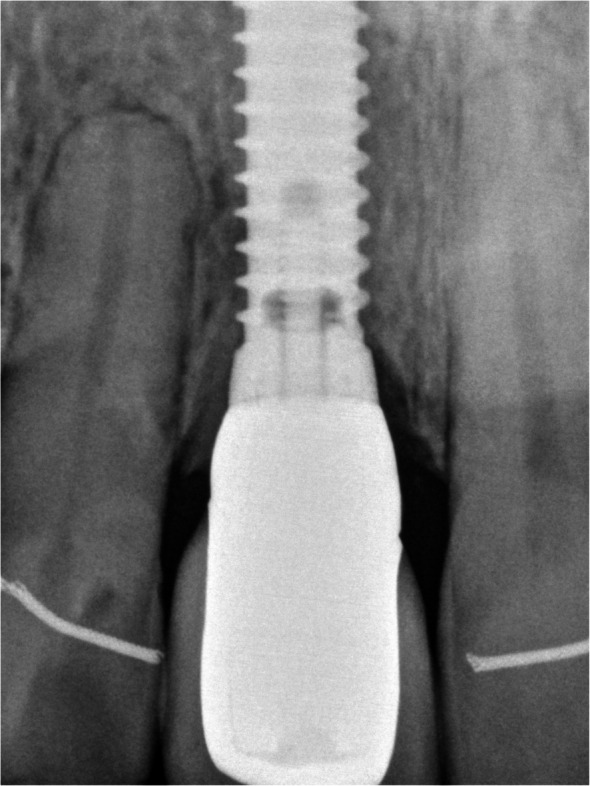
Postoperative x-ray after osseointegration

In addition to the beagle dog histology provided by Hurzeler [1], Schwimer et al. [2] provided human histology showing bone formation between the remaining dentin of the socket shield and the implant surface. Whilst this histology was made possible due to a failed implant, it needs to be noted that this was an unintentional socket shield, and hence socket-shield dimensions as well as height reduction might have been less than desirable with regard to the here described socket-shield technique and therefore contributed to the implant failure.

This literature review examines the available evidence regarding the socket-shield technique as postulated by Prof. Hurzeler.

A recently published systematic review [3] concluded that modifications to the socket-shield technique as postulated by recent studies was associated with promising results. Furthermore, it was stated that the choice of graft materials for socket-shield application did not play much of a role. However, data presented in the review by Mourya et al. does not seem to either confirm or oppose this statement. Therefore this critical review was conducted.

## Material and methods

### Study procedure and material

This study aims to collect and evaluate the present knowledge with regard to the socket-shield technique as described by Hurzeler et al. [1].

The following inclusion and exclusion criteria were applied:

Inclusion criteria:

- Studies including case reports investigating the socket-shield technique
- Studies published in English
- Studies published between January 01, 1990, and May 12, 2019

Exclusion criteria:

- Animal studies
- In vitro studies
- Literature reviews
- Studies published in languages other than English

### Search strategy

This literature review was performed accordingly to the PRISMA 2009 checklist.

A PubMed database search (www.ncbi.nlm.nih.gov/pubmed) was conducted to identify relevant publication.

The following search term including Boolean operators was used:

(dental AND ((implant OR implants) AND ((socket shield OR socket-shield OR root membrane OR Huerzeler OR partial extraction therapy))). This returned 288 positive results, all abstracts were scrutinised, and articles found to meet the inclusion and exclusion criteria were downloaded for further investigation and screened by both authors independently.

Furthermore, the bibliographies of all downloaded articles were screened manually to identify further relevant studies.

**Fig. 14:**
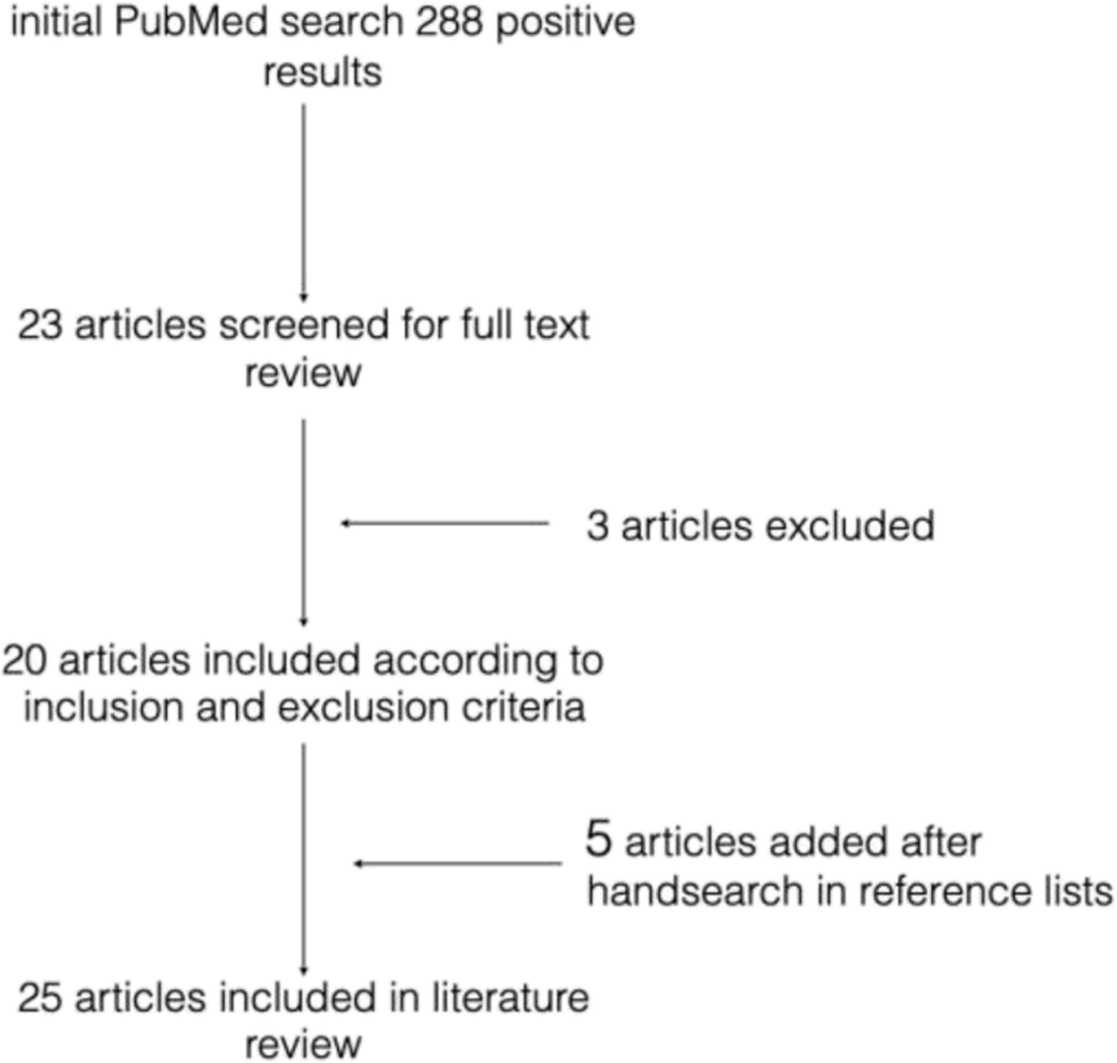
Flowchart search strategy

In addition, a Google Scholar search with the identical search phrase was conducted to identify further potentially relevant articles. Studies found in addition to the PubMed database search were labelled hand search (Fig. 14).

### Data extraction

Data pertinent to the use of the socket-shield technique was extracted and entered into the master table (Table 1).

**Table 1:** Included studies

*n*	Author	Title	Year	Study type	*N* patients	*n* implants	Region	Augmentation	Observation period	po radiography supplied	Follow-up radiography supplied	Osseointegration rate	Complications	*n* survival implants	Cosmetic outcome	Results/conclusion
19	Bramanti, et al. [4]	Postextraction dental implant in the aesthetic zone, socket shield technique versus conventional protocol	2018	Randomised controlled trial	40	40	13–23 or 33–43	allograft (copiOs)	36			100%	Nil	100%	PAS significantly higher in test group	Significantly higher PAS and lower amount of crestal bone change in test group
10	Dary et al. [5]	The socket shield technique using bone trephine: a case report	2015	Case report	1		Pre molar (maxilla)		0				Not reported			Authors conclude that socket-shield represents a promising technique to preserve buccal bone
23	Arabbi et al. [6]	Socket shield: a case report	2019	Case report	1	2	Teeth 21 and 11	No	Nil	No	No	100%	Not recorded	n/a	Not recorded	Authors conclude that the socket-shield technique has not enough clinical data to recommend for daily practice
11	Baumer et al. [7]	The socket-shield technique: First histological, clinical and volumetrical observation after separation of the buccal tooth segment- a pilot study	2013	Case report	1 post IV bisphosphonate use	2	Canine (maxilla) -Socket shield central incisor -No socket shield	No	Nil	No	No		Not reported	2		Socket-shield technique is technique sensitive and needs for more scientific data Socket-shield technique can still not be generally recommended for clinicians in daily practice. Yet the observed results are promising
12	Baumer et al. [8]	Socket shield technique for immediate implant placement—clinical, radiographic and volumetric data after 5 years	2017	Retrospective clinical study	10 (5 male, 5 female)	Unknown	Unknown		51 to 63 months (mean 51 months)			100%	Not reported		Volumetric changes measured by means of stl comparison Mean loss of buccal tissue − /0.37 ± 0.18 mm avr mid facial recession − .33 ± .23 mm Mean loss of marginal bone level 0.33 mm ± 0.43 mm (mesial) 0.17 ± 0.36 mm at distal Pink aesthetic score mean 12 (11–14)	Authors conclude, scientific evidence lacking, socket shield suggests advantages in immediate implant placement, low morbidity and favourable cost-benefit ratio additionally might provide more predictable aesthetic outcome in complex cases Further research required for long-term stability
5	Cherel and Etienne [9]	The socket-shield technique and immediate implant placement	2013	Case report	1	2	Central incisors	Bio-Oss	6 months post restoration	No	1 month post restoration 6 months post restoration		Not reported	2		PA at follow-up shows no interpret bone change
4	Dayakar et al. [10]	Immediate implant combined with modified socket-shield technique: a case letter	2018	Case report	1	1		Unknown	3 months	Yes	pa 2 months		Not reported	1		Authors conclude that SS-technique is successful in preserving of tissue
24	Dayakar et al. [10]	The socket-shield technique and immediate implant placement	2018	Case report	1	1	Tooth 22	No	Nil	No	No	100%	Nil	n/a	Not recorded	Authors conclude that socket-shield technique shows promising result
25	Glocker et al. [11]	Ridge preservation with modified “socket-shield” technique: a methodological case series	2014	Case report	3	3	13 (2) 22 (1)	Yes (Bio-Oss) (2) fgg (1)	Nil	Yes	No	100%	Not reported	n/a	Not recorded	Authors conclude that the socket-shield technique is a cost-effective technique which avoids resorption of bundle bone
13	Gluckman et al. [12]	A retrospective evaluation of 128 socket-shield cases in the esthetic zone and posterior sites: partial extraction therapy with up to 4 years follow-up	2018	Retrospective study	Unknown	128	Numerous	Unknown	1–4 years	na	na	123/128 (96.1%)	5 implant failures, reason unknown 3 infected socket shields + mobile removal of socket shield, retention of implant 2 socket shields mobile, removal of socket shield and implant 12 internal socket shield exposures 4 external (oral cavity) exposures of socket shields 2/4 external exposures required ctg 1 socket shield migration	123	Author noted that no dark hues or recession exposing the abutment to fixture interface were noted	Similar osseointegration rate compared to traditional treatment concept, with the added benefit of a less invasive approach. Most common complication—internal exposure of socket shield—conclusion that the ss was not reduced enough to all for adequate space, furthermore authors now recommend the ss reduction to bone level
18	Gluckman et al. [13]	The pontic-shield: partial extraction therapy for ridge preservation and pointed site development.	2016	Case report	10	14	Anterior maxilla	ctg, xenograft, fgc	12–18 months				1 socket shield exposure			Subjective observation noticed tissue volume to be preserved 1 patient had complications—all 3 socket shields exposed due to failure of soft tissue closure Authors note that limited scientific evidence for this technique nomenclature is noted as being inconsistent Authors note that additional research and scrutiny is needed to validate this technique for use in daily clinical practice
21	Guo et al. [14]	Tissue preservation through socket-shield technique and platelet-rich fibrin in immediate implant placement	2018	Case study	1	1	Tooth 21	Yes—PRF	18 months	Yes	Yes	100%	None	1	Stable soft tissue reported	The socket-shield was effective in preserving the peri-implant tissue and contour
20	Han et al. [15]	The modified socket shield technique	2018	Clinical trial	30	40	Premolar, canine and incisors in mandible and maxilla	No	1 year po	n/a	n/a	100%	None	40	Not supplied	Authors conclude that the socket shield technique is safe and efficient in preserving bone
3	Huang et al. [16]	The root membrane technique: human histologic evidence after 5 years of function	2017	Case report	1	1		Bio-Oss	9 months		cbct		Not reported	1	Score 13	
14	Hurzeler et al. [1]	The socket-shield technique: a proof-of-principle report	2010	Proof of concept/case report	1	1	Central incisor maxilla	Emdogain	0	No	No		Not reported			Author concludes that this case report supports socket shields as a viable implant placement concept. This technique potentially could be used to reduce the risk of resorption of the bundle bone post extraction.
6	Kan et al. [17]	Proximal socket shield for interplant papilla preservation in the aesthetic zone	2014	Case report	1	1	Central incisor	Bio-Oss + puros (allograft) CTG	1 year post restoration	Yes	pa 1 year		Not reported	1		Authors report satisfactory aesthetic result, but that the socket shield is a technique sensitive procedure with limited long-term evidence
2	Mitsias et al. [18]	Clinical benefits of immediate implant socket shield technique	2017	Case report	1	1		None	5 years				Not reported	1		Buccal bone plate was maintained, no evidence or resorption apical and medial part between socket shield and implant was filled with mature bone coronal part that was connective tissue
16	Mitsias et al. [19]	A step-by-step description of PDL-mediated ridge preservation for immediate implant rehabilitation in the esthetic region	2015	Case report	1	1	Central incisor maxilla	Not stated	3 years	Yes	Yes		None	1		Novel technique similar to the socket shield technique (difference is the direct implant to root fragment contact) Authors report that this technique might prevent psychological implications of tooth extraction ( as part of root remains); however, a careful case selection is recommended
17	Szmukler-Moncler et al. [20]	Unconventional implant placement part III: implant placement encroaching residual roots—a report of 6 cases	2014	Case report	6	6	Molars mandible, premolars maxilla and mandible, central incisor maxilla	Not stated	3–9 years	Yes	Yes	6/6	1 case possible resorption of tooth fragment 1 implant with crestal bone loss to second/third thread 9 years post restoration	5–1 patient drop out		Author reports that the presence or absence of root-filling material seemed to have no effect on implant on outcome
7	Nevins et al. [21]	Late dental implant failure associated with retained root fragments: case report with histologic and SEM analysis	2018	Case report	2	2	1st molars	Case 1: bio-Oss Case 2: DFDBA	Case 1: 8 + years Case 2: 4 years	Case 1: yes Case 2: yes	Yes		Case 1: advanced peri-implantitis, root fragment attached to messiah aspect evident Case 2: loss of integration	0		Case 1: Human histology (LM) revealed implant in bone contact consistent with osseointgration, graft biomaterial in close proximity to fixture, direct implant contact to cementum of the retained root surface, no sign of periodontal ligament Case 2: LM shows bone in between implant surface and root fragment late implant failure might contribute to unintentionally remaining root fragments
1	Pour et al. [22]		2017	Case report	1	1		None	3 months				Not reported	1		Authors conclude that no added cost for patient, single surgical procedure, reduced morbidity, possibility of tx in patient with previous end pathology tutors describe as favourable technique for dental practice
8	Schwimer et al. [2]	Human histologic evidence of new bone formation and osseointegration between root dentin (unplanned socket-shield) and dental implant: case report	2018	Case report	1	1	Pre molar	Unknown	2 years	No	No		Loss of integration peri-implantitis	0		Authors reported failed osseointegration 2 years post restoration, human histology revealed root fragment attached to implant, bone formation on implant surface evident absence of fibrovascular tissue.
15	Siormpas et al. [23]	Immediate implant placement in the esthetic zone utilizing the “root-membrane” technique: clinical results up to 5 years postloading	2014	Retrospective case series	46 (20 male 26 female)	46	Anterior maxilla	Nil	24 –60 months (mean 40 months(	na	na	100%	1 case resorption of root fragment	46		Pre-, post-operative cbct in 4 cases with maintained buccal bone volume in 3/4 cases Author concluded that similar complication rate to traditional placement protocol but minimising of facial bone volume changes Author concludes bone volume has remained stable; however, volumetric investigation using cbct data was only carried out in 4/46 cases.
22	Siormpas et al. [24]	The root membrane technique: a retrospective clinical study with up to 10 years of follow-up	2018	Retrospective clinical study	182	250	Anterior	No	Mean 49 months	n/a	n/a	Not supplied	Not reported	5 (87.9%)	Not recorded	Author reports similar success rate as in conventional immediate implants
9	Wadhwani et al. [25]	Socket shield technique: a new concept of ridge preservation	2015	Case report	1	1	Central incisor	Yes, material unspecified	0	Yes	No		Unknown	Unknown	Unknown	Authors conclude that this case report suggest alveolar bone preservation

## Results

The initial database search returned 229 results. After screening the abstracts, 23 articles were downloaded and further scrutinised. Twelve studies were found to meet the inclusion and exclusion criteria. The reference lists were further subjected to a hand search which returned a further 6 studies for this literature review (Fig. 14).

The studies included are summarised in Table 1.

### General overview

Hurzeler et al. published the first article on the socket-shield technique [1]. Since then, the amount of publications has steadily increased, with the largest number of publication in 2018 (Table 2). Most publications were case reports; however, retrospective studies have been published as early as 2014. Retrospective studies make up the minority of data published (Table 3). Prospective studies have not been cited to date.

**Table 2:** Publications on socket-shield technique

Year of publication	***n*** publications	Case report/retrospective study
**2010**	1	1/0
**2013**	2	2/0
**2014**	3	2/1
**2015**	3	3/0
**2016**	1	1/0
**2017**	3	2/1
**2018**	4	3/1

**Table 3:** Study type of published studies

Study type	***n***
**Randomised clinical trial**	1
**Case report**	20
**Retrospective study**	3
**Clinical trial**	1
**Total**	25

### Type of publications

The majority of publications identified in this literature review were case reports (16/24) [1, 5–7, 9–11, 13–23, 25–27]. Three publications were retrospective clinical trials/studies [8, 12, 24]; one publication was a randomised clinical trial [4].

### Cohort size

The cohort size did vary considerably, whilst the majority of case reports reported on single clinical cases up to 3 cases. The three retrospective clinical trials did report on as many as 128 cases followed up [12] and as little as 10 [8].

Only one randomised clinical trial was identified in this literature review [4] with a total of 40 implants in 40 patients and a follow-up period of 36 months.

### Observation time

The observation time reported did vary considerably from 0 months up to 9 years [20]. The majority of publications however did not state observation times past 1 year.

### Outcome

All studies reported on osseointegration of implants and reported osseointegration rates comparable to traditional placement protocols. Generally, the case reports identified in this literature review reported an osseointegration rate of 100%. However, both referred to retrospective clinical trials (Gluckman et al. [12], Siormpas et al. [24]) reporting significantly lower osseointegration rates of 96.1% and 87.9%.

The only randomised clinical trial (Bramanti et al. [4]) identified on the other hand reported 100% osseointegration; however, the cohort size was only 40 implants for both test and study group combined.

Six studies did report additional to this regarding the cosmetic outcome [8, 10, 12, 23].

Several studies/case reports reported on the cosmetic outcome of the implant treatment; however, the cosmetic outcome was not consistently evaluated, one study used the pink aesthetic score, one study simply mentioned the positive outcome, and one study employed volumetric measurements to disciple the amount of tissue remodelling [25].

### Preservation of buccal architecture/bone-height

Almost all of the studies presented reported on the preservation of the alveolar ridge and/or soft tissue buccal to the implant [1, 4, 5, 7, 8, 10–14, 16, 17, 19, 22, 23, 25, 26].

However, the reporting was inconsistent with regard to how this outcome was measured.

Three studies analysed the volumetric changes by means of 3-dimensional scans [7, 8, 23], one study evaluated the buccal bone by means of taking post-operative CBCT scans [5], whereas others used the pink aesthetic score [4, 16], and finally, some studies did not specify how the outcome was measured at all [1, 10–14, 17, 19, 22, 25, 26] and merely stated a good outcome was achieved.

### Complications

Six out of 18 studies reported on possible complications with the socket-shield technique [12, 13, 20, 23].

The exposure (internal and/or external) of the socket shield as reported by Gluckman et al. [12] was the most commonly reported complication pertinent to the socket-shield technique with a total of 17 exposed socket shields reported. Gluckman et al. [12] reported 12 internal and 4 external shield exposures. Two of the external exposures required a connective tissue graft to achieve closure, and three infected socket shields required removal of the socket shield altogether; however, the implants were able to be retained.

The remaining complications reported were resorption of the socket shield (2), peri-implantitis (2), non-integration of implants, or failed implant integration (7).

## Discussion

The majority of publications identified relating to the socket-shield technique are clinical case reports and are unfortunately of little scientific value.

Therefore, the “Discussion” section will mainly focus on four clinical trials identified in the literature [4, 8, 12, 24] as well as publications by Hurzeler et al. [1] due to its impact as proof of concept, and Mitsias et al. [18] and Schwimer et al. [2] as they represent the only available human histologies to date.

In general, cohort size in the clinical trials varied significantly. Gluckman et al. [12] reported a large cohort of 128 implants followed up over a significant period of up to 9 years which has weighted influence on the data presented in this literature review. The remaining trials had very small cohorts and short observation times.

Hurzeler et al. [1] first reported the socket-shield technique as a proof of concept in an animal model. Whilst they were able to demonstrate the formation of a bony layer between the socket shield and the implant surface through histological evaluation, the animal model poses limitations when the technique is translated to humans.

Mitsias et al. [18] and Schwimer et al. [2] demonstrated similar outcomes.

The article by Bramanti et al. [4], whilst of small cohort size and short observation period, constituted the only randomised clinical trial to date in literature. However the surgical protocol in this study did vary from the technique described by Hurzeler et al. [1] in so far as the implant preparation was performed with the tooth root in place, which was split just prior to implant placement. Bramanti et al. [4] furthermore were the only study group concluding that bone graft in combination with the socket-shield technique is mandatory. This is in direct contrast to Hurzeler et al. [1] who concluded that an advantage of the socket-shield technique would be the fact that bone grafting with its cost and added complexity is not required.

With regard to clinical evaluation of the socket-shield technique, only Baumer et al. [8] reported on volumetric changes affecting the buccal tissues complex. Siormpas et al. [23] evaluated radiographic changes affecting the remaining root fragment, whilst Gluckman et al. [12] focused exclusively on clinical complications.

Bramanti et al. [4] did report the pink aesthetic score.

Therefore, inconsistent use of reporting measures across the studies severely limited comparison of results.

Surprisingly, as the vast majority of socket-shield implants reported placed were in the cosmetic zone, use of a relevant and consistent method of evaluation such as a pink aesthetic score, or more preferably determination of volumetric changes, was found to be rare.

The study by Baumer et al. [8], which was the only study to evaluate volumetric changes, reported only subtle facial tissue changes when compared to conventional immediate implant placement and restoration techniques.

Whilst their results were encouraging and showed similar, if not superior outcomes to conventional treatment protocols, the small cohort size limits what conclusions can be drawn.

Siormpas et al. [23] on the other hand used radiographs exclusively to assess bone changes following implant placement. Consequently, assessment was limited to a 2-dimensional analysis of space changes. Given that the rationale behind the socket-shield technique is to preserve buccal volume after implant placement, and that this is not discernible from conventional two-dimensional radiographs, this manuscript provides very limited evidence supporting the technique.

Gluckman et al. [12] reported low complication rates; the most common adverse outcome reported was the exposure of the root fragment either internally ( towards the implant restoration) or externally (exposure towards the buccal soft tissue). The authors reported that neither of these complications were difficult to manage or caused an adverse aesthetic outcome.

## Conclusion

Whilst the socket-shield technique potentially offers promising outcomes, reducing the need for invasive bone grafts around implants in the aesthetic zone, clinical data to support this is very limited. The limited data available is compromised by a lack of well-designed prospective randomised controlled studies. The existing case reports are of very limited scientific value. Retrospective studies exist in limited numbers but are of inconsistent design. At this stage, it is unclear whether the socket-shield technique will provide a stable long-time outcome.

Hence, caution is advised at this stage when using the socket-shield technique in routine dental practice. Clinicians are advised to exercise best clinical judgement when considering to use the socket-shield technique for treatment.

Further clinical studies, preferably prospective randomised controlled clinical trials involving power analysis to determine an adequate cohort size to inform statistical interpretation which would allow conclusions to be drawn, are desirable.

## Data Availability

The dataset(s) supporting the conclusions of this article is available in PubMed.
